# Proceedings of the Saudi Health Simulation Conference 2018

**DOI:** 10.1186/s41077-018-0065-6

**Published:** 2018-04-23

## A1 The impact of simulation-based training on teamwork and communication in the Emergency Department: a pre-test post-test evaluation

### Aida Darweish^1^, Ali Alshareef^1^, Shayma Millibary^2^, Shahad Bafakeer^3^, Malak BinShihon^3^, Leen Othman^3^

#### ^1^Emergency Department, Jeddah, Saudi Arabia; ^2^Quality Department, National Guard Health Affairs Hospital (NGHA), Jeddah, Saudi Arabia; ^3^College of Medicine, King Saud Bin Abdulaziz University for Health Sciences, Jeddah, Saudi Arabia

**Background:** Poor communication and teamwork in the Emergency Department (ED) carries high safety risk for patients. A strategy to prevent or minimize medical errors is by simulation-based teamwork and communication training. This study provided simulation-based training to the ED staff in the National Guard Health Affairs hospital (NGHA), Jeddah, Saudi Arabia. The TeamSTEPPS curriculum was used to improve teamwork, communication, and reduce medical errors.

**Methods:** This is a single-subject experimental design research with the intervention incorporating simulation-based training in ED cases. The study focused on 3 domains. 1) Patient safety in the ED, 2) Inter-professional and multidisciplinary simulation team training, 3) Team dynamic enhancement by using TeamSTEPPS principles. There were three phases: 1) A pre-intervention perception survey using T-TPQ (TeamSTEPPS - Teamwork Perceptions Questionnaire), 2) 18 multidisciplinary full-day sessions through simulation that were followed by focused brief on site sessions (FBOS) in the ED on a weekly basis, 3) Post-intervention staff perception assessment using T-TPQ (Fig. 1).

**Fig. 1 (abstract A1).**
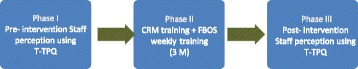
See text for description

**Results:** The survey covered five different aspect of teamwork including: Team Function, Leadership, Situation Monitoring, Mutual Support and Communication. Overall staff perceptions of teamwork in the ED: the response in pre-test was 69% (*n* = 1850) “Agree”, 27% (*n* = 724) “Neutral” and 4% (*n* = 121) “Disagree”. The overall response improved significantly post-test with the *p*-value <0.0001. “Agree” increased by 15% to be 80% (*n* = 3058), “Neutral” decreased by 36% to be 17% (*n* = 653), and “Disagree” response decreased by 39% to be 3% (*n* = 104) (Fig. 2).

**Conclusion:** The results showed improvement in perceptions of improved teamwork and communication behaviour among ED staff. This is likely to have led to avoidance of medical errors. A feature of the training was follow up in the clinical environment. A further phase of utilizing TEAM assessment tools in the real ER environment will be conducted to further confirm the effectiveness of simulated-based training that is integrated with clinical practice.

**Fig. 2 (abstract A1).**
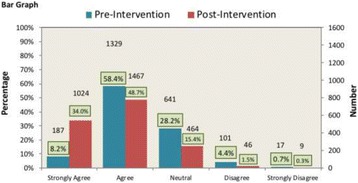
Respondents’ ratings in Perceptions of Teamwork Questionnaire (*n* = 1850). * The research IRB approval has been taken from the KAIMARC (King Abdullah International Medical Research Centre)

**References**

1. Graban, M. (2016). Lean hospitals: improving quality, patient safety, and employee engagement: CRC Press.

2. King, H. B., Battles, J., Baker, D. P., Alonso, A., Salas, E., Webster, J., Toomey, L., Salisbury, M. (2008). TeamSTEPPS^TM^: team strategies and tools to enhance performance and patient safety. In: Henriksen, K., Battles. J.B., Keyes, M.A., Grady, M.L., editors. Advances in Patient Safety: New Directions and Alternative Approaches (Vol. 3: Performance and Tools). Rockville (MD): Agency for Healthcare Research and Quality (US); 2008 Aug.

3. Cooper, S., Cant, R., Porter, J., Sellick, K., Somers, G., Kinsman, L., & Nestel, D. (2010). Rating medical emergency teamwork performance: development of the Team Emergency Assessment Measure (TEAM). *Resuscitation, 81*(4), 446-452.

## A2 Healthcare simulation in Jordan: current situation and future vision

### Fadwa N. Alhalaiqa

#### Faculty of Nursing**,** Philadelphia University, Amman, Jordan

**Objective:** To review published literature on issues related to the use of healthcare simulation in Jordan.

**Methods:** Although simulation is increasingly being developed and deployed worldwide to train healthcare personnel (Ahmed, et al., 2010), in Jordan it is still not fully developed and faces many challenges. This paper reviews published literature on any issues with regards to use of simulation among health care providers in Jordan in order to identify the current situation and future vision. MEDLINE, CINAHL, ERIC, EBSCO, Scopus, Psychinfo and PubMed databases were searched for research papers published up to December 2017 that identified simulation use among healthcare providers in Jordan. Reference lists of related articles were also searched for descriptive quantitative and qualitative studies.

**Results**: Ten studies were identified, of which nine met inclusion criteria. Eight studies used experimental designs to determine the effect of simulation (Akhu-Zaheya, Gharaibeh, & Alostaz, 2013; Al-Refaie, Fouad, Li, & Shurrab, 2014; Aqel & Ahmad, 2014; Basheti, 2014; Tawalbeh, 2016; Tawalbeh & Tubaishat, 2014; Toubasi, Alosta, Darawad, & Demeh, 2015; Tubaishat & Tawalbeh, 2015). Six studies were conducted by researchers with a nursing background and two by pharmacists. One study investigated attitudes and perceptions of students toward simulations. All experimental studies reported significant results and improvement in skills, knowledge acquisition, knowledge retention, and self-efficacy and confidence among simulation users (Akhu-Zaheya, et al., 2013; Al-Refaie, et al., 2014; Aqel & Ahmad, 2014; Basheti, 2014; Tawalbeh, 2016; Tawalbeh & Tubaishat, 2014; Toubasi, et al., 2015; Tubaishat & Tawalbeh, 2015). Jordanian nursing students’ attitudes and perceptions were generally in favor of simulation.

**Conclusion**: Use of simulation among healthcare providers (particularly nursing) increased their knowledge and skills and very likely resulted in increased quality of care. Although there are promising results of simulation use in Jordan, it is not well developed. This may be associated with many challenges (e.g. high cost, lack of teamwork approach, lack of policy maker support, curriculum reform challenges, financial support, and infrastructure, manpower, and information technology capabilities) (Doolen, et al., 2016; Hosny, Johnston, Pucher, Erridge, & Darzi, 2017). Substantive efforts are needed to resolve these challenges and to craft a strategy to use simulation in the near future; particularly in the current situation in terms of the increasing numbers of health students, and quality and safety focusing on ethical and moral rights and, cost-benefit analysis based practice.

**References**

1. Ahmed, K., Keeling, A. N., Fakhry, M., Ashrafian, H., Aggarwal, R., Naughton, P. A., et al. (2010). Role of Virtual Reality Simulation in Teaching and Assessing Technical Skills in Endovascular Intervention. *Journal of Vascular and Interventional Radiology, 21*(1), 55-66.

2. Akhu-Zaheya, L. M., Gharaibeh, M. K., & Alostaz, Z. M. (2013). Effectiveness of Simulation on Knowledge Acquisition, Knowledge Retention, and Self-Efficacy of Nursing Students in Jordan. *Clinical Simulation in Nursing, 9*(9), e335-e342.

3. Al-Refaie, A., Fouad, R. H., Li, M.-H., & Shurrab, M. (2014). Applying simulation and DEA to improve performance of emergency department in a Jordanian hospital. *Simulation Modelling Practice and Theory, 41*(Supplement C), 59-72.

4. Aqel, A. A., & Ahmad, M. M. (2014). High-Fidelity Simulation Effects on CPR Knowledge, Skills, Acquisition, and Retention in Nursing Students. *Worldviews on Evidence-Based Nursing, 11*(6), 394-400.

5. Basheti, I. A. (2014). The Effect of Using Simulation for Training Pharmacy Students on Correct Device Technique. [Article]. *American Journal of Pharmaceutical Education, 78*(10), 1-10.

6. Doolen, J., Mariani, B., Atz, T., Horsley, T. L., Rourke, J. O., McAfee, K., et al. (2016). High-Fidelity Simulation in Undergraduate Nursing Education: A Review of Simulation Reviews. *Clinical Simulation in Nursing, 12*(7), 290-302.

7. Hosny, S. G., Johnston, M. J., Pucher, P. H., Erridge, S., & Darzi, A. (2017). Barriers to the implementation and uptake of simulation-based training programs in general surgery: a multinational qualitative study. *Journal of Surgical Research, 220*(Supplement C), 419-426.e412.

8. Tawalbeh, L. I. (2016). Effect of Simulation on the Confidence of University Nursing Students in Applying Cardiopulmonary Assessment Skills: A Randomized Controlled Trial. *The Journal Of Nursing Research: JNR*.

9. Tawalbeh, L. I., & Tubaishat, A. (2014). Effect of simulation on knowledge of advanced cardiac life support, knowledge retention, and confidence of nursing students in Jordan. *The Journal Of Nursing Education, 53*(1), 38-44.

10. Toubasi, S., Alosta, M. R., Darawad, M. W., & Demeh, W. (2015). Impact of simulation training on Jordanian nurses’ performance of basic life support skills: A pilot study. *Nurse Education Today, 35*(9), 999-1003.

11. Tubaishat, A., & Tawalbeh, L. I. (2015). Effect of Cardiac Arrhythmia Simulation on Nursing Students’ Knowledge Acquisition and Retention. [Article]. *Western Journal of Nursing Research, 37*(9), 1160-1174.

## A3 Comparison between simulation–based and didactic lecture-based medical ethics teaching for third year dental students in Princess Nourah University – a pilot study

### Haseena Fuad

#### Department of Preventive Dentistry, College of Dentistry, Princess Nourah University, Riyadh, Saudi Arabia

**Background:** Although simulation-based teaching is a stable feature of ethics education, little is known about the attributes of the scenarios that make them effective. Emotions are an inherent part of ethical decision-making and scenario-based learning has potential to evoke emotion while offering relevant knowledge. This is likely to facilitate learning.

**Objective:** The aim of this study is to compare simulation–based and didactic lecture-based medical ethics teaching for third year dental students.

**Methods:** This qualitative study was conducted to expose third year dental students to two methods of education for medical ethics, a traditional didactic lecture and a standardized patient/simulation-based scenario, which focused on concepts of professional ethics, communication skills, and law; and to assess which method is more effective. The students were given traditional didactic lectures on principles of medical ethics, communication skills and doctor–patient relationship. A simulation-based scenario with standardized patients was created and performed for the students. Students had to make ethical decisions after the scenario. Efficacy of each teaching method was compared through use of multiple choice questionnaires (MCQ). Student satisfaction was evaluated by means of a questionnaire.

**Results:** Thirty-five students responded to the survey measuring the efficacy and satisfaction of simulation-based learning. Simulation proved more resource intensive requiring specialized equipment for making ethical decisions with self-confidence. Twenty one (60%) students agreed that simulation-based learning improved the ability to assess ethical issues. Sixteen (46%) students agreed that they gained communication skills to engage in difficult conversations with patients and by-standers.

**Conclusion:** Simulation-based training can provide a bridge between didactic and observational learning to clinical practice by allowing repetitive practice, and mastery-based learning prior to or in parallel with traditional bedside training. Although simulation (especially standardized-patients) is used to teach professional ethics, communication skills, and law, it has not been used to teach dental students about ethical conflicts and/or to assess their understanding of ethical principles applied to difficult clinical decision-making.

**References**

1. Orsolya Solymos, et al. Pilot study comparing simulation-based and didactic lecture-based critical care teaching for final-year medical students. BMC Anesthesiology 2015;15:153.

2. Chase E. Thiel, Shane Connelly, Lauren Harkrider, et al. Case-Based Knowledge and Ethics Education: Improving Learning and Transfer Through Emotionally Rich Cases. Sci Eng Ethics 2013;19:265–286.

3. Poom Tritrakarn, Benjamin W. Berg, Richard T. Kasuya, Damon H. Sakai. Can We Use Simulation to Teach Medical Ethics? Hawaii Journal of Medicine & Public Health 2014;73(8):262-264.

## A4 Best practices of using simulation in high-stakes clinical assessments

### Loui Alsulimani

#### Department of Emergency Medicine, King Abdulaziz University, Riyadh, Saudi Arabia

**Background:** Assuring the competency of healthcare providers (HCPs) is a critical function of training and accrediting institutions. Using simulation in high-stakes assessment has been evolving as a method to enhance and improve the assessment process. There is a concurrent need to address challenges and establish best practices to assure the best quality for high-stakes assessments.

**Objective:** The aim of this study is to present best practices of using simulation in high-stakes clinical assessment described in literature to provide guidance for stakeholders who are interested in such applications.

**Methods:** This review of literature searched for articles discussing challenges, best practices and future directions of using simulation in high-stakes assessment. Studies included common modalities (standardized patients, high-fidelity mannequins, part-task trainers, virtual simulation and hybrid simulation). The search covered the following databases: PubMed, Education Resource Information Center (ERIC), Cumulative Index to Nursing and Allied Health Literature (CINAHL), and the Cochrane library.

**Results:** The initial screening for simulation assessment in the databases resulted in 19,292 articles. After the application of a refining search strategy, 40 articles were included for comprehensive evaluation. Most of the articles gave recommendations regarding best practices of implementing simulation in high-stakes assessments. Best practices were classified (based on the stage of the implementation) into three categories: planning, during the assessment and post-assessment (Table 1).

**Conclusion:** The use of simulation for high-stakes assessment is promising. Assuring the application of best practices driven by previous experiences described in the literature is likely to assure the highest quality activities.

**Table 1 (abstract A4).** Classification of best practices based on the stage of assessment implementation

Phase	Examples of best practices
Planning phase	Right modality of simulation for the tasks to be evaluated
	Real practice design to reflect the highest possible fidelity
	Appropriate structure and resources standards of the assessment centre
	Proper security measures to prevent breach of the exam content
	Clear and user friendly scenarios for SPs, examiners and technical support staff
	Determine the metrics of the assessment
	Carefully chosen/designed assessment tools (checklists, global ratings) to facilitate high standard metrics
	Raters should be trained and qualified based on a preset protocol
	Measures to assure high reliability and validity e.g. increasing the number of scenarios, designing task-specific stations and standardizing the exams administration
	Targeted metrics, e.g. Kappa >0.75, reliability, validity, accessibility, feasibility
During the assessment phase	Timing each station 5-10 minutes, in acute care medicine can be shortened to 5 minutes
	Video and audio recording, as a quality measure, to be considered
	Synchronizing and standardizing timing of the exam conducted in multiple centres
	Availability of real-time technical support in all centres
	Reinforcing measures to prevent cheating
	Room designation during the examination, i.e. examiner, SP and/or technician
Post-assessment phase	Evaluation of the assessment process
	Psychometric analysis of the scores to support the validity of the assessment
	Explore for confounding factors that influence the outcome
	Review the issues that appeared during the exam and actions accordingly
	Develop continuous improvement plan
	Update manuals and/or protocols
	Demonstrate results and appeal process to the learners
	Conduct remediation process as planned before

## A5 Different methods for role-play using peers as simulated patients in achieving medical communication skills

### Abdul Sattar Khan, Rabel Khawaja, Jihan Hakeem, Mohammad Bastaweesy, Juliet Balaes, Romina Labaniego

#### College of Medicine, King Faisal University, Riyadh, Saudi Arabia

**Background:** Role-play is used to enhance communication skills (CS) in medical students and has been proven to offer a level of pragmatism when incorporated with technical skills training, which lead to improved patient-doctor interaction. In order to achieve the CanMED competency, the *communicator,* we have small group teaching on CS at our College. It comprises role-play among peers with simulated patients who are students chosen from each CS group. We use two types of role-play, *Round Robin* and *Relays or Carousel* method. The Round Robin method includes three students; a doctor, a patient and an observer and Relay method comprises four doctors, four observers – one for each doctor - and a patient. This study explored the effectiveness of different methods of role-play to support the development of CS by working with peer students as simulated patients.

**Methods:** The study was conducted in May 2017 at College of Medicine, King Faisal University, Al-Ahsa. All first-year students and trainers were included in the study. A structured questionnaire was distributed among students and trainers at the end of the teaching block. The comparison of two methods was evaluated at the end of all sessions. The questionnaire included 15 statements related to the role-play methods based on Likert scale ranging from 1=strongly disagree to 5=strongly agree. Questions were formulated from a previous study however; the study had a different context and method. Therefore, the questions were modified and more items were added by CS experts based on the teaching of CS sessions and according to the competencies relevant to CS. Data was collected and entered to SPSS version 20.00. Descriptive statistics were computed for all the items. Wilcoxon signed-rank test was applied to compare different items for both methods and to obtain *p*-value. A *P*-value of <0.05 was considered as significant.

**Results:** A total of 246 students and trainers participated in the study. The results of statistical test for comparing both methods suggest that there is significant difference in Round Robin and Relay method for most of the questions *P*-value < 0.05. However, there was no significant difference on role-play as an interesting mode of learning (*P*-value= 0.062 (CI=0.052–0.061)), role-play generates better attention span (*P*-value=0.138 (CI=0.129–0.142)) and role-play helps in developing self-confidence (*P*-value=0.178 (CI=0.170 – 0.185)).

**Conclusion:** Overall, the results suggest that there is a difference between role-play methods and students and trainers perceive the Round Robin method as more effective.

## A6 Undergraduate nursing students’ satisfactions with simulation experience: a survey-based study

### Sumayah Kamal Fatane^1^, Christine Ateah^2^, Nicole Harder^2^, Edward Giesbrecht^3^

#### ^1^Department of Nursing, Renal Sub-Committee, Ministry of Health, Makkah, Saudi Arabia; ^2^Faculty of Health Sciences, College of Nursing, University of Manitoba, Winnipeg, Canada; ^3^Department of Occupational Therapy, University of Manitoba, Winnipeg, Canada

**Background:** The literature of simulation in healthcare education indicates insufficient information regarding the effect of different levels of simulation fidelity on student satisfaction.

**Objectives:** The study was conducted to evaluate students’ perceptions of Low-Fidelity Simulation (LFS) and High-Fidelity Simulation experiences (HFS).

**Methods:** A descriptive, cross-sectional, retrospective post-test study was approved by the Education/Nursing Research Ethics Board (ENREB). Fourth-year nursing students at the University of Manitoba, enrolled in fall term 2014 were invited to complete a survey electronically using Fluid Survey. The survey includes the Satisfaction with Simulation Experience Scale (SSE), and a ranking question. The SSE was developed by Levett-Jones et al. (2011), and consists of three subscales: Debriefing and Reflection (D&R); Clinical Reasoning (CR); and Clinical Learning (CL). The data input and analyses were conducted using SPSS version 22.

**Results:** Thirty-five eligible students participated. A paired t-test analysis revealed a significant difference in CL subscale means between LFS (M=4.09) and HFS (M=3.78), *p* = 0.008. The ranking question revealed that the opportunities to practice new skills, and to apply clinical reasoning and decision-making were among the top three ranked features for both LFS and HFS. Also the LFS’s preparation and orientation and HFS’s engagement and realism were top ranked.

**Discussion:** The findings from the SSE provides evidence that students valued simulations of low and high fidelity, and that clinical learning was found to be preferable for LFS. Clinical learning is enhanced and transferred to clinical practice through practice in engaging and realistic environments, and by reflection on this practice during debriefing [1, 3]. The top ranked “apply new skills,” for both low and high fidelity simulations suggests that simulation could provide safe and controlled environments to practice. The students highly ranked “apply CR and DM,” for both simulations indicates the students were able to think through the case scenarios and provide appropriate care. The students top ranked LFS “orientation and preparation”. Also, they ranked highly HFS's “engagement and realism,” which suggest the authentic learning environment in HFS allows engagement. Recommendations includes combining the HFS with LFS, support and training for instructors, more student orientation to, and additional HFS sessions. Collecting data retrospectively on students’ experiences may have affected their responses, and further research using different design and larger sample is required. This study can add another dimension to the costly technology-advanced HFS if preferable to LFS activities, or whether incorporate components of both.

**References**

1. Howard, V. M., Englert, N., Kameg, K., & Perozzi, K. (2011). Integration of simulation across the undergraduate curriculum: Student and faculty perspectives. Clinical Simulation in Nursing, 7(1), e1-e10. doi:10.1016/j.ecns.2009.10.00

2. Levett-Jones, T., McCoy, M., Lapkin, S., Noble, D., Hoffman, K., Dempsey, J., Arthur, C., &Roche, J. (2011). The development and psychometric testing of the Satisfaction with Simulation Experience Scale. Nurse Education Today, 31(7), 705–710. doi:10.1016/j.nedt.2011.01.004.

3. Reed, S. J., Andrews, C. M., & Ravert, P. (2013). Debriefing simulations: Comparison of debriefing with video and debriefing alone. Clinical Simulation Nursing, 9(12), e585-e591. doi: org/10.1016/j.ecns.2013.05.007 in

## A7 Impact of mock code simulation program in improving paediatric residents’ resuscitation skills

### Tarek Hazwani, Zahra Alhassan, Abdullah Yaqub, Mohannad Antar

#### King Abdullah Specialist Children's Hospital King Abdulaziz Medical City, Ministry of National Guard Health Affairs, Riyadh, Saudi Arabia

**Background:** King Abdullah Specialist Children's Hospital, King Abdulaziz Medical City recruits 20-25 paediatric residents yearly. Before 2015, paediatric residents were not involved in Code Blue events. Eight-four per cent graduate without running a code during their training. This will likely have a profound impact on their first Code Blue event as a senior resident (Code leader). Although the rate of paediatric Code Blue is low (9 codes in 2016), the residents’ chances to run a code is low. Impact on clinical outcome has been demonstrated in many studies that observed increased survival rates correlated with increased number of mock codes [1-4].

**Objectives:** To evaluate our program to enable residents to manage simulated Code Blue in situ.

**Methods:** Our goal was to help Code teams learn. We conducted unscheduled simulation Codes in real settings. Each resident led one simulated Code during second-year, and one during their third-year of residency. Evaluation data was collected in each Code for the team leader including knowledge assessment, and Critical Recourse Management (CRM) score. Scores were analysed to evaluate improvements after the second Code. Other data related to team members’ performance was also collected, including: CPR initiation time and first epinephrine dose timing.

**Results:** A pilot group of six paediatric residents involved in simulated Codes twice, with 12±3 month intervals, revealed improvement in knowledge and CRM scores (Fig. 1). Data also showed the adherence to AHA guidelines was 33% in paediatric codes for team leaders not involved in simulation, and 100% for those who underwent simulation. Other data showed CPR initiation time by first nurse responder improved from an average of 58 seconds in first opportunity to 30 seconds in the fifth opportunity.

**Discussion:** Results showed improvement in paediatric residents’ performances in simulated Codes by their second session. These simulation sessions have also had a positive impact on other team members’ performance and patient safety including AHA guidelines adherence and CPR initiation time. Although there are limitations with our small sample size and the measurement tools, this simple evaluation points to the key role of simulation in supporting residents in managing a Code Blue in paediatric settings.

**References**

1. Allan CK, Thiagarajan RR, Beke D, Imprescia A, Kappus LJ, Garden A, et al. Simulationbased training delivered directly to the pediatric cardiac intensive care unit engenders preparedness, comfort, and decreased anxiety among multidisciplinary resuscitation teams. J Thorac Cardiovasc Surg. 2010;140(3):646–52.

2. Andreatta P, Saxton E, Thompson M, Annich G. Simulationbased mock codes significantly correlate with improved pediatric patient cardiopulmonary arrest survival rates. Pediatr Crit Care Med. 2011;12(1):33–8.

3. Thomas EJ, Taggart B, Crandell S, Lasky RE, Williams AL, Love LJ, et al. Teaching teamwork during the Neonatal Resuscitation Program: a randomized trial. J Perinatol. 2007;27(7):409–14.

4. Thomas EJ, Williams AL, Reichman EF, Lasky RE, Crandell S, Taggart WR. Team training in the neonatal resuscitation program for interns: teamwork and quality of resuscitations. Pediatrics. 2010;125(3):539–46.

**Fig. 1 (abstract A8).**
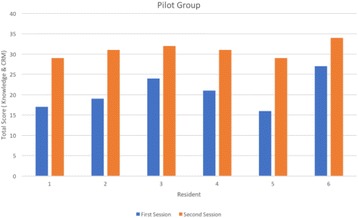
Total scores for paediatric residents from first to second sessions (*n* = 6)

## A8 Using virtual reality as an educational tool for communication and collaboration competencies among undergraduate medical students at King Saud bin Abdulaziz University for Health Sciences Jeddah, Saudi Arabia: an experimental study

### Lama Ibrahim Sultan

#### King Abdul-Aziz Medical City, Jeddah, Saudi Arabia

**Background:** Undergraduate medical education is constantly evolving with curricular shift to be competency-based. According to SaudiMED, *communication* and *collaboration* is the core domain [1]. Literature shows that repeated practice followed by feedback is mandatory for behavioral changes. We live in an era of “digital natives” generation. Different technologies can be used to support their experiential learning [2] such as Virtual Reality (VR).

**Methods:** Experimental study was conducted where 4th year medical students participated in a workshop to address the core competence. They were divided into two groups according to the educational tool: group one received 360 VR videos and group two received lectures. The outcome factors were all quantitative variables: Perception level (a questionnaire was given before the session), MCQs score (20 MCQs pre- and post-session to assess knowledge retention), OSCE score (to assess skill acquisitions), satisfaction level (a questionnaire was given after VR session). All the 169 medical students were included in the study.

**Results:** The response rate was 88% for 169 participants, 57 (VR) and 112 (Lecture). Majority of students (93%) think that VR can be used in medical education. Post MCQs score (out of 20) was significantly higher in VR group when compared to lecture group (17.4±2.1 vs. 15.9±2.9, *p*-value <0.001). The OSCE score was also better with VR group (12.9±4.1 vs. 9.8±4.2, *p*-value <0.001). Overall rating of VR satisfaction experience showed a mean of 7.26 out of 10.

**Conclusion:** VR provides a rich, interactive, engaging educational context, thus supporting experiential learning-by doing. In fact, it raises interest and motivation for student and effectively support knowledge retention and skills acquisition.

**References**

1. Zaini RG, Bin Abdulrahman KA, Al-Khotani AA, Al-Hayani AM, Al-Alwan IA, Jastaniah SD. Saudi Meds: a competence specification for Saudi medical graduates. Med Teach 2011;33(7):582-4.

2. Kolb DA. Experiential learning: Experience as the source of learning and development. New Jersey, NJ: Prentice-Hall; 1984.

## A9 Development of a new tool for self-assessment of confidence, expectations/satisfaction and performance for cupping therapy simulation training modules

### Tamer Aboushanab, Mohammed Khalil, Ahmed El-Olemy, Saud AlSanad

#### National Center for Complementary and Alternative Medicine, Ministry of Health, Riyadh, Saudi Arabia

**Background:** Simulation is considered a safe training method. Simulation training technology is a popular method of learning for healthcare professionals worldwide [1]. It includes performing a medical procedure on simulators to increase the confidence and skills of trainees before conducting the procedures on humans [2]. Cupping therapy *(Hijama)* is a widely used traditional healing therapy which is performed by applying cups on selected body points by sucking air to induce sub-atmospheric pressure inside the cup either by heat or suction [3]. The use of simulation in cupping therapy training was an innovation developed by National Center for Complementary and Alternative Medicine (NCCAM), Ministry of Health (MOH), Saudi Arabia [4]. There is no available validated evaluation tools for assessment of cupping simulation learning due to the novelty of this method. An internal committee of NCCAM, Saudi Arabia developed this questionnaire, as a part of the evaluation process of their training courses.

**Objectives:** To discuss the development of a new tool which can be used for evaluation of cupping therapy simulation-based learning.

**Methods:** Fifteen items in the questionnaire were divided into three scales. The three scales were confidence, expectations/satisfaction, and performance. Each scale included five questions and used 5 Likert scale responses from 1 to 5 [Fig. 1]. Statistical Package for Social Sciences (SPSS) Software Version 20 was used for data entry, management and analysis. Internal reliability of the questionnaire and scales was evaluated by Cronbach's alpha test.

**Results:** 50 healthcare professionals participated in the study. They were selected from the trainees of the simulation courses provided by NCCAM as a part of the program’s evaluation. Cronbach's alpha of items deleted were ranged from 0.91 to 0.92 for each item. Cronbach's alpha of confidence scale was 0.85, expectations/satisfaction scale was 0.81, performance scale was 0.94, and total evaluation was 0.92. These values showed good internal consistency of the scales.

**Conclusion:** Cupping Simulation Training Evaluation Questionnaire (CSTEQ) is a promising new tool for evaluating self-reporting of confidence, expectations/satisfaction and performance of trainees. It may be used as a tool for improvement of cupping simulation training programs. Further large scale trials, and validation studies should be conducted.

**Fig. 1 (abstract A9).**
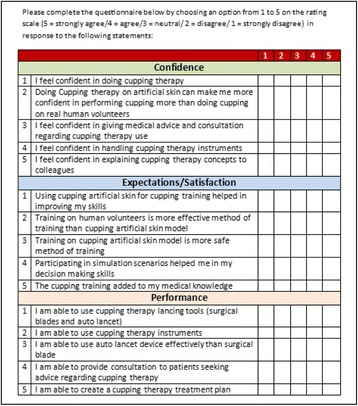
See text for description

**References**

1. Alinier, G., Hunt, B., Gordon, R., & Harwood, C. (2006). Effectiveness of intermediate-fidelity simulation training technology in undergraduate nursing education. Journal of advanced nursing, 54(3), 359-369.

2. Kunkler K. The role of medical simulation: an overview. The International Journal of Medical Robotics and Computer Assisted Surgery. 2006 Sep 1;2(3):203-10.

3. Arslan M, Gökgöz N, Dane Ş. The effect of traditional wet cupping on shoulder pain and neck pain: A pilot study. Complementary therapies in clinical practice. 2016 May 31;23:30-3.

4. Aboushanab, T., & AlSanad, S. M. (2017). Simulation In Cupping Training: An Innovation Method. J Acupunct Meridian Stud 2017;10(6):409e410

## A10 The current status of using simulation based learning in integrative and complementary medicine training programs

### Tamer Aboushanab, Saud AlSanad

#### National Center for Complementary and Alternative Medicine, Ministry of Health, Riyadh, Saudi Arabia

**Background:** Simulation-based learning in medicine and surgery is a training approach where new practitioners can practice and acquire clinical skills in a safe, risk free environment similar to the real clinical situation [1]. The history of using simulation in the field of complementary medicine is dated to 1027 when Ancient Chinese physician Wang Wei-Yi used two bronze real-size statutes for teaching acupuncture and surface anatomy [2].

**Objectives:** The aim of this review is to give a brief overview of the current status of simulation-based learning in integrative and complementary medicine training programs.

**Methods:** The relevant literature published in English prior to December 2017 was retrieved from PubMed, Cochrane, and ScienceDirect databases to identify articles on the use of simulation in integrative and complementary medicine training programs. Two reviewers evaluated the results based on predefined inclusion, and exclusion criteria.

**Results:** Seven articles met inclusion criteria. A virtual reality simulator for acupuncture training providing trainees’ with realistic feeling of touch was developed [3]. The phantom acupoint tool significantly improved students’ manipulation skills in acupuncture simulation training [4]. Virtual reality haptic back (VHB) was designed by two colleges (osteopathic and engineering) in Ohio University to help in the training of osteopathic students and related therapies in clinical palpatory diagnosis method [5, 6]. A hospital based massage learning course was developed and offered by Midwest academic medical centre, USA. The simulation-based practice in the simulation centre was a basic part of the course [7]. Chapman et al. showed that using simulation in the training of cervical spine manipulation for early trainees of chiropractic was beneficial. Early trainees acquired the basic skills initially and gained the confidence to proceed to training on real patients [8]. Cupping therapy simulation learning was an innovative idea developed by the National Center for Complementary and Alternative Medicine (NCCAM), Ministry of Health, Saudi Arabia (Fig. 1) [9].

**Discussion:** Benefits of using simulation-based learning were reported in the field of integrative and complementary medicine training. Haptic simulation was used in the fields of acupuncture (Fig. 2), massage, osteopathy, and chiropractic learning. However, the part-task mannequin simulators were used in cupping therapy training and cervical manipulation as a part of chiropractic training. We recommend introducing simulation-based learning in the field of complementary medicine training to help trainees gain confidence and basic clinical skills. Future large scale studies to evaluate the simulation based learning programs and trainees’ performance are encouraged.

**Conclusion:** This review identified the introduction of simulation-based learning in five integrative and complementary therapies which were: acupuncture, osteopathy, massage, chiropractic, and cupping therapy.

**Fig. 1 (abstract A10).**
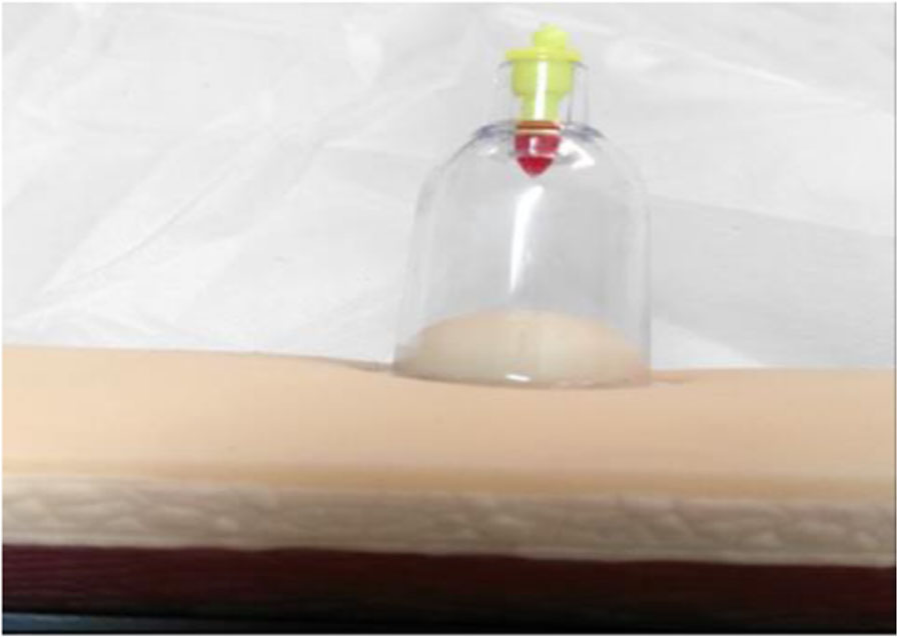
Cupping Therapy part-task trainer

**Fig. 2 (abstract A10).**
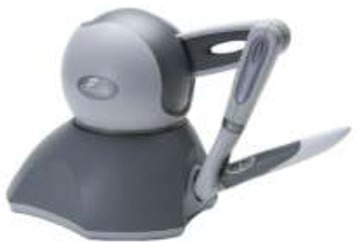
The PHANTOM® Omni™ model represents acupuncture needle, to train on puncturing technique on VR environment

**References**

1. Khunger N, Kathuria S. Mastering surgical skills through simulation-based learning: Practice makes one perfect. *Journal of cutaneous and aesthetic surgery*. 2016 Jan;9(1):27.

2. Owen, H. (2012). Early use of simulation in medical education. *Simulation in healthcare*, *7*(2), 102-116.Heng, P-A., Tien-Tsin Wong, Rong Yang, Yim-Pan Chui, Yong Ming Xie, Kwong-Sak Leung, and Ping-Chung Leung. “Intelligent inferencing and haptic simulation for Chinese acupuncture learning and training.” *IEEE Transactions on Information Technology in Biomedicine* 10, no. 1 (2006): 28-41.

3. PloS one, 10(2), e0117992.

4. Howell, John N., Robert R. Conatser, Robert L. Williams, Janet M. Burns, and David C. Eland. “The virtual haptic back: a simulation for training in palpatory diagnosis.” *BMC medical education* 8, no. 1 (2008): 14.

5. Williams II, Robert L., Mayank Srivastava, John N. Howell, Robert R. Conatser Jr, David C. Eland, Janet M. Burns, and Anthony G. Chila. “The virtual haptic back for palpatory training.” In *Proceedings of the 6th international conference on Multimodal interfaces*, pp. 191-197. ACM, 2004.

6. Dion, Liza J., Susanne M. Cutshall, Nancy J. Rodgers, Jennifer L. Hauschulz, Nikol E. Dreyer, Barbara S. Thomley, and Brent Bauer. “Development of a hospital-based massage therapy course at an academic medical center.” *International journal of therapeutic massage & bodywork* 8, no. 1 (2015): 25.

7. Chapman, Peter D., Norman J. Stomski, Barrett Losco, and Bruce F. Walker. “The simulated early learning of cervical spine manipulation technique utilising mannequins.” *Chiropractic & Manual Therapies* 23, no. 1 (2015): 23.

8. Tamer Aboushanab, Saud M. AlSanad, Simulation in Cupping Training: An Innovation Method, *Journal of Acupuncture and Meridian Studies*, 2017, 10.1016/j.jams.2017.10.003.(http://www.sciencedirect.com/science/article/pii/S200529011730170X)

## A11 Pressure ulcer prevention in the intensive care unit: applying CRESENT system integration model

### Hani Lababidi^1,2^, Fadi Shehadeh^3^, Shadi Almozainy^2^, Mohamad Almani^2^

#### ^1^CRESENT, King Fahad Medical City, Riyadh, Saudi Arabia; ^2^Pulmonary & Critical Care Department, King Fahad Medical City, Riyadh, Saudi Arabia; ^3^Nursing Education Department, King Fahad Medical City, Riyadh, Saudi Arabia

**Background:** The incidence of pressure ulcers in ICU ranges from 8.8% to 23% [1]. System integration utilizes the principle of system engineering and risk management to improve patient care [2]. CRESENT system integration model uses the acronym of CRESENT to describe the various stages of system integration.

**Objectives:** The aim of this study is to apply the newly described CRESENT system integration model to the prevention of pressure ulcers in ICU.

**Methods:** The steps for CRESENT system integration model include: **C**larify, **R**eview, **E**xamine, **S**imulate, **E**xecute, **N**otify and **T**rack. This model is applied for a project on prevention of pressure ulcers in ICU at KFMC.

**Results:** The CRESENT model is applied as follows: 1) **C**larify the problem that prompted system integration: Head of ICU is concerned about an increase in pressure ulcers rate. 2) **R**eview of the current data shows pressure ulcers rate in ICU around 10 pressure ulcers/1000 patient days, while the target is < 5 (Fig. 1). 3) **E**xamine the possible causes and identifies areas of improvements through simulation. 4) **S**imulate by using system modeling to develop applicable SBE activity. An 8-hour SBE workshop on pressure ulcer prevention in ICU is developed by a simulation educator (FS). 5) **E**xecute: A total of 92 inter-professional simulation (IPS) are conducted between March and April 2017. Around 82 (60%) ICU staff nurses attended these activities. 6) **N**otify the stakeholders (Chairman of Critical Care Department) through detailed report on the SBE activities, attendance, evaluations and assessment. 7) **T**rack the impact of the system integration by following the preset indicator(s) to evaluate the outcome of the SBE. The rate of pressure ulcer was reduced by 50% from the 1^st^ & 2^nd^ quarters of 2017 (Fig. 1).

**Discussion:** System integration is an effective modality to identify areas of potential threats and improvement strategies. The SBE activity on prevention of pressure ulcer in ICU provided knowledge and skills to the ICU staff, as well as defined responsibilities and collaboration among the treating team in ICU. The following categories of healthcare givers are trained together: Nurses, physicians, respiratory therapists, physiotherapists, wound care team, pharmacists, risk management and dietitians.

**Conclusion:** The CRESENT model for system integration provides a framework that can be used to apply the principles of risk management in health care facilities.

**Fig. 1 (abstract A11).**
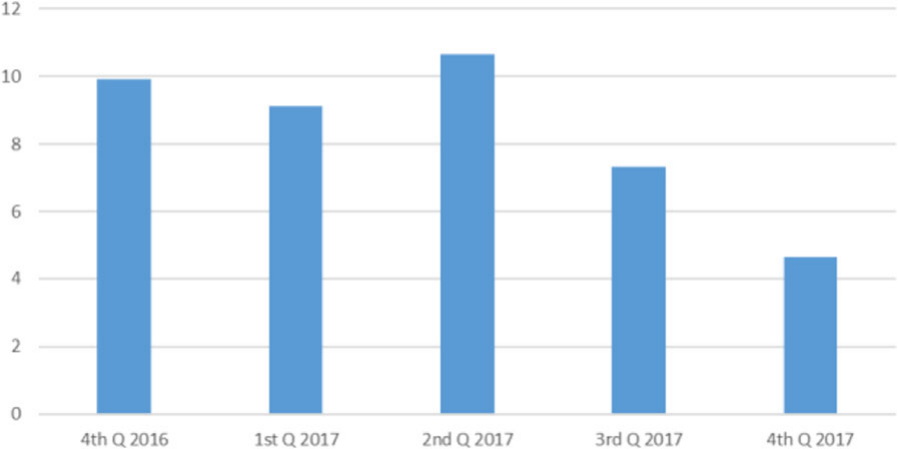
Pressure ulcer rate in ICU 2017

**References**

1. Krupp AE, Monfre J. Pressure ulcers in the ICU patient: an update on prevention and treatment. Curr Infect Dis Resp. 2015 Mar;17(3):468.

2. William Dunn, Ellen Deutsch, Juli Maxworthy, Kathleen Gallo, Yue Dong, Jennifer Manos, Tiffany Pendergrass, Victoria Brazil. Systems Integration. In: Adam I. Levine, Samuel DeMaria Jr., Andrew D. Schwartz and Alan J. Sim (eds.) The Comprehensive Textbook of Healthcare Simulation 10.1007/978-1-4614-5993-4 © Springer Science + Business Media New York 2013.

## A12 Identifying priorities, needs and difficulties of standardized patients in clinical skill and simulation center, King Abdulaziz University, KSA

### Khalid Alatawe, Mohammed Almalki, Abdulaziz Boker

#### Clinical Skills & Simulation Center, King Abdulaziz University, Jeddah, Saudi Arabia

**Background**: Standardized Patient (SP) methodology is used as teaching tool in students’ sessions and various training workshops, as well as evaluation technique in Objective Structured Clinical Examinations (OSCEs) and high-stake assessments. The Clinical Skills & Simulation Center at King Abdulaziz University established an SP program in collaboration with other medical departments. We recruited many SPs for various cases and scenarios of different genders and age categories. The increasing number of SPs enabled us to create key performance indicators to evaluate mechanisms that affect the program in terms of priorities and the needs and difficulties faced by program participants.

**Objectives:** This study aimed to identify priorities, needs and difficulties faced by participants in SP program.

**Methods:** SPs were surveyed through bilingual structured interviews and a questionnaire. The interviews were designed for children, SPs with language barriers and who are unable to read. The questionnaire has three parts: 1) Demographics: age, gender, education, social and marital status; 2) Focused questions: waiting time, period inside exam, scenario performance, exam environment, feeling of inconvenience effects, ranking of needs, and willingness to participate again; 3) Open-ended questions.

**Results:** A total of 64 SPs were surveyed during academic year 2016-2017. The survey revealed 89% of SPs were willing to participate in future events, and 81% were willing to engage in volunteer activities. Most SPs (92%) thought that the exam scenarios were clear while 28% thought the waiting time was too long. Fourteen percent of SPs had post-event negative feelings which were effectively relieved with post-event debriefing and reassurance. The most significant needs and priorities of the SPs are presented in Fig. 1. Financial rewards, providing means of transportation, type of examination, and the time commitment needed were the top-rated priorities.

**Conclusion:** Financial rewards exceeded all priorities. The SP program should develop protocols and a hospitable environment to meet and prepare SPs prior to conduct teaching and examination encounters. We recommend further study to develop the most practical options for SPs recruitment, safety and recurrent participation.

**Fig. 1 (abstract A12).**
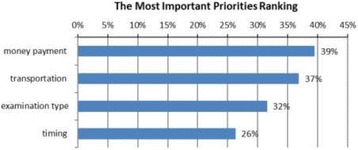
SPs ratings of facets of their practice (*n* = 64)

## A13 Peer simulated debriefing training: a descriptive study to advance debriefing skills in small group feedback

### Abdullah Almarshed^1^, Shadi Almoziny^1^, Charles Pozner^2^, Jeffrey Cooper^3^, Dara O’keeve^4^, Steven Yule^2^, Roxane Gardner^3^, Jenny Rudolph^3^, Usamah Al-Zoraigi^1^

#### ^1^CRESENT, King Fahad Medical City, Riyadh, Saudi Arabia; ^2^STRATUS, Brigham & Women’s Hospital, Harvard University, Boston, MA, USA; ^3^Center for Medical Simulation (CMS), Boston, MA, USA

**Background:** Debriefing can be defined as an activity that follows a simulation experience led by a facilitator wherein feedback is provided on the simulation participants’ performance while positive aspects of the completed simulation are discussed and reflective thinking encouraged (National League for Nursing, 2008). Peer simulated debriefing is a technique developed by the authors to further advance debriefing skills in small group feedback.

**Objective:** The aim of this study is to introduce a formally structured training exercise to practice debriefing techniques between peers through role playing.

**Methods:** Peer simulated debriefing training was initiated by three of the authors (AA, SA and UA) and developed through focus groups with simulation experts. Forms were designed to help run formal structured sessions. A dry run was applied with detailed feedback from experts in simulation and pedagogy. Sessions were conducted at STRATUS, Brigham and Women’s Hospital and Center of Medical Simulation (CMS), Boston, USA. The Debriefing Assessment of Simulation in Healthcare (DASH) tool was used to provide quantitative measures of debriefing quality.

**Results:** The peer simulated debriefing training is an exercise that takes 90 to 110 minutes depending on the number of participants. It includes assigning two learners to watch a video recording of a simulation scenario twice. The learners are asked to pick up key actions based on agreed objectives, assume frames for debriefing and document these actions on a special forms. The two learners will assign and play the role of important characters in the scenario. A third learner joins the group and practice pre-briefing, introduction of the session and building a safe environment. The whole team then watches the video once again followed by a debriefing. After the debriefing, the actors reflect on the debriefing by describing their feeling and debriefing the debriefer. An expert watches the whole process and gives final feedback.

**Conclusion:** Simulated debriefing training allows peers to practice debriefing in a safe environment with regular feedback**,** promotes training on scenario analysis and identifies learning gaps, provides an opportunity for participants to practice “debriefing the debriefing” method and presents a structured method that tracks participants’ progress and areas for development.

## A14 The feasibility of using the Grounded Theory to analyze qualitative mock code data

### Usamah Al-Zoraigi^1,2^, Charles Pozner^2^

#### ^1^CRESENT, King Fahad Medical City, Riyadh, Saudi Arabia; ^2^STRATUS Center for Medical Simulation, Brigham & Women’s Hospital, Harvard Medical School, Boston, MA, USA

**Background:** Unannounced simulated mock codes are used to assess the readiness of medical teams and the institution to manage cardiopulmonary arrests [1]. Large amounts of quantitative and qualitative data are usually collected. The analysis and meaningful translation of qualitative data from mock codes can be challenging. Grounded theory can provide a framework to generate concepts through analysis of qualitative data from mock codes [2].

**Objective:** The aim of this study was to determine the feasibility of using Grounded Theory to analyze data generated from mock codes.

**Methods:** The mock code program at the Brigham & Women Hospital is sponsored by the Emergency Response Committee. Locations are selected on a rotational basis to ensure uniform assessment of the three separate code teams that respond to designated areas. Two mock codes per month are conducted without forewarning of time or location; the majority during normal working hours. Each mock code is followed by 5-7 minutes of debriefing. A report of the drill is provided to local and hospital lead clinicians and administrators. Quantitative data are collected such as time to arrival of first responder, time to first chest compression, and so on. Qualitative data is collected by a single faculty member who leads the mock codes and/or as reported by the respondents during the debriefing. This includes administrative and clinical protocol deviations, quality of CPR, articulation of an exit strategy, equipment failures, staffing deficiencies, misuse of equipment, access issues among others. All debriefing comments and observations were clustered by one of the authors (UA) based on similarity. We stratified the data into two broad categories: training gaps (gaps to be addressed by educational interventions) and systems gaps (gaps to be addressed through changes in processes).

**Results:** One hundred and fifty sessions with 994 debriefing comments and observations were collected between September 2003 and July 2015. After exclusion of 96 positive comments, of the 898 gaps identified, 777 (87%) were related to training gaps and 121 (13%) were related to system gaps. The frequency of these comments are plotted over time (Fig. 1). We identified a decrease in the frequency of training gaps over time, while there was no change in the system gaps.

**Fig. 1 (abstract A14).**
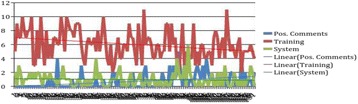
Plotting of qualitative data from mock codes between 2003 and 2015.

**Discussion:** Employing Grounded Theory, we were able to cluster performance gaps of observed, unannounced mock codes into two categories: training-related gaps and system-related gaps [3, 4]. We found that over time, educational gaps decreased but there was no change in the frequency of system-related gaps. Based on this, allocation of resources to improve system-related performance gaps appears to be indicated.

**Conclusion:** Grounded Theory can be used to analyze the qualitative data generated from mock codes and can provide administrators information on strategies to improve key performance indicators (KPIs).

**References**

1. Herbers MD, Heaser JA. Implementing an in Situ Mock Code Quality Improvement Program. Am J Crit Care 2016;25(5):393-9.

2. Glaser B, Strauss L. Discovery of grounded theory for qualitative research: Chicago: Aldine; 1967.

3. Frank J. Villamaria MD. Using Simulation to Orient Code Blue Teams to a New Hospital Facilities. Sim Healthc 2008;3(4):209–216.

4. Iirola T, Lund VE, Katila AJ, Mattila-Vuori A, Palve H. Teaching hospital physicians’ skills and knowledge of resuscitation algorithms are deficient. *Acta Anaesthesiol Scand* 2002;46:1150 –1154

## A15 Simulation-based teaching and didactic lecture in gaining and retaining knowledge among undergraduate medical students: a randomized controlled trial

### Marwa M. R. Tawfik^1,2^, Amel A. Fayed^1^, Amal F. Dawood^1^, Gehan H. Ibrahim^3^, Eman Al Mussaed^1^

#### ^1^College of Medicine, Princess Nourah bint Abdulrahman University, Riyadh, KSA; ^2^Hepatobiliary Unit, Internal Medicine Department, Alexandria University, Alexandria, Egypt; ^3^Medical Biochemistry Department, Faculty of Medicine, Suez Canal University, Ismailiia, Egypt

**Background:** Most medical teaching is still delivered by traditional face-to-face interaction [1]. However, simulation-based teaching (SIM) is growing as an effective technique in medical education which can be used as alternative to lectures [2].

**Objectives:** The aim of our study was to evaluate the effectiveness of SIM versus traditional lectures in improving and retaining knowledge.

**Methods:** A randomized controlled trial was conducted among 72 medical students at Princess Nourah bint Abdulrahman University (PNU). Using random number sequence, students were randomized into two groups, 36 each. Each group received the same scientific information about the diagnosis and management of bronchial asthma by the same instructor but through different teaching techniques. In one group the instructor used didactic lecture with video recording while in the other group, mannequin simulation with role-play session was used. Knowledge testing immediately before and after the teaching sessions and 4 months later was done using 30 multiple-choice questions. A questionnaire was distributed to students to assess their satisfaction with the teaching methods.

**Results:** There was no significant difference between the two groups regarding their scores in the pre-test; the simulation group scored 41.2±10.6 and the lecture group scored 38.8±7.2, *p*-value =0.3. The simulation group scored higher than the lecture group in the post-test and in the second post-test, however, this difference was not statistically significant (*p*-value =0.50 and 0.40 respectively). Both groups showed an improvement in the average score from the pre-test to post-test but the improvement in the simulation group was higher than that in the lecture group (8.4±10.7 and 7.1±11.8 respectively) though this difference was not statistically significant (*p*-value =0.61). Additionally, students in the simulation group were significantly more satisfied by the teaching modality than students in the lecture group as reflected by the higher satisfaction score (42.4± 11.7 versus 29.7±9.3, *p*-value <0.01)

**Conclusions:** SIM was as effective as the didactic lecture in immediately improving and retention of knowledge. SIM is a more satisfactory and interesting way of teaching as reported by students. If it is integrated in undergraduate program, it may help to overcome obstacles of clinical training and the lack of proper medical students’ exposure to real patients because of ethical considerations.

**References**

1. Bhatti I, Jones K, Richardson L, Foreman D, Lund J, Tierney G. E-learning vs lecture: which is the best approach to surgical teaching? Colorectal Disease: the official journal of the Association of Coloproctology of Great Britain and Ireland. 2011;13(4):459-62.

2. Maddry JK, Varney SM, Sessions D, Heard K, Thaxton RE, Ganem VJ, et al. A Comparison of Simulation-Based Education Versus Lecture-Based Instruction for Toxicology Training in Emergency Medicine Residents. Journal of Medical Toxicology. 2014 05/21;10(4):364-8. PubMed PMID: PMC4252281.

## A16 Medical health simulation awareness and opinion among Saudi Commission of Health Specialty Trainees in Saudi Arabia

### Ahmed Hesham Ibrahim^1^, Sawsan Alyousef^1,2^, Meshal Saleh Alduhaim^1^

#### ^1^Specialized Children Hospital, King Fahad Medical City, Riyadh, Saudi Arabia; ^2^CRESENT, King Fahad Medical City, Riyadh, Saudi Arabia

**Background:** Simulation is used to create a better and safer environment for healthcare practitioners and to decrease medical errors. The Saudi Commission for Health Specialties (SCFHS) has introduced multiple simulation courses in specialties as part of the training curricula for residents and fellows.

**Objectives:** The aim of this study is to assess awareness of medical simulation among SCFHS trainees and identify obstacles that prevent them from participating in such activities.

**Methodology:** Cross sectional study through electronic survey to residents and fellows under SCFHS from regions in Saudi Arabia: Riyadh, Jeddah, Eastern Province, Jizan and Northern Region. Only complete surveys were considered for analysis. We excluded BLS, ACLS, PALS & ATLS courses.

**Result:** A total of 313 SCFHS trainees responded to the electronic survey, response rate was 35%. The majority are 25-30 years of age (Fig. 1). Residents counted for 285 (91%) (R2=30% & R3=22%) and fellows for 28 (9%) (F1=2.2% & F2=1.6%). The specialties of the participating SCFHS trainees are presented in Fig. 2. One hundred and fifty-one (48.2%) had heard of medical simulation, while 87 (28%) had used simulation. The majority of the trainees who are involved in simulation belong to governmental institutes 72 (83%), 15 (17%) to university institutes and none to private institutes. Around two-thirds of the trainees have simulation centers at their institution. The trainees believe medical simulation should be mandatory in undergraduate and postgraduate training, 83% and 93% respectively. The perceptions of simulation are presented in Table 1 and the obstacles in Table 2.

Most common courses suggested by non-surgical trainees are airway management, central line insertion under ultrasound guidance, lumbar puncture and crisis management. While laparoscopy and basic surgical skills are suggested by surgery residents and postpartum hemorrhage by obstetric/gynecology residents

**Conclusion:** Medical simulation is still not widely practiced in the training of SCFHS trainees in various specialties.

**Fig. 1 (abstract A16).**
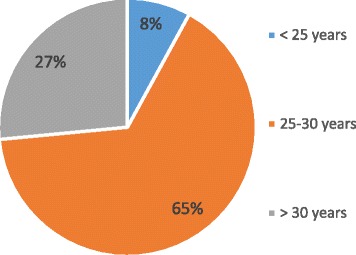
Age groups of participating SCFHS Trainees

**Fig. 2 (abstract A16).**
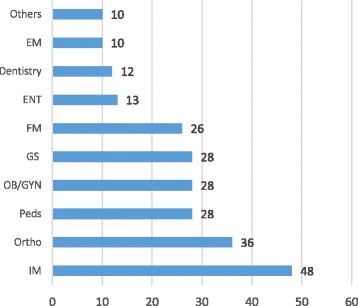
Specialty of Participating SCFHS Trainees

**Table 1 (abstract A16).** Perceptions of SCFHS trainees about medical simulation

Perceptions	Yes	No	*P*-value
Don’t know where simulation can be used	56 (64%)	31 (36%)	< 0.001
Lack of knowledge of benefit of simulation	41 (47%)	46 (53%)	0.448
Simulation is not suitable	24 (28%)	63 (72%)	< 0.001
Heard negative experience from others	8 (9%)	79 (91%)	< 0.001
Can improve outcome of patient care	85 (98%)	2 (2%)	< 0.001
Can improve physician skills	84 (97%)	3 (3%)	< 0.001
Can improve physician medical knowledge	82 (94%)	5 (6%)	0.029
Can improve team work	84 (97%)	3 (3%)	< 0.001
Skills simulation courses should be repeated frequently	83 (95%)	4 (5%)	0.006

**Table 2 (abstract A16).** Perceived obstacles by SCFHS trainees about medical simulation

Perceived Obstacles	Yes	No	*P*-value
Lack of time to attend or create simulation models	58 (67%)	29 (33%)	< 0.001
Lack skilled staff in simulation development	42 (48%)	45 (52%)	0.649
Lack of knowledge on how to create simulation courses	41 (47%)	46 (53%)	0.448
Lack of simulation equipment	39 (45%)	48 (55%)	0.172
Lack of time for simulation activities	1 (1%)	86 (99%)	< 0.001
Cost of simulation courses	58 (67%)	29 (33%)	< 0.001
Cost of simulation equipment	55 (63%)	32 (37%)	< 0.001
Fees for simulation instructors	29 (33%)	58 (67%)	< 0.001

## A17 Does debriefing after simulation-based scenarios improve non-technical skills for healthcare teams in the emergency department?

### Abdullah AlMarshed^1^, Emad Masuadi^2^, Henk Schmidt^2^, Mohamud Mohamud^2^, Hani Lababidi^1^, Amani Azizalrahman^1^, Usamah Alzuraigi^1^, Asim Alsaeed^1^

#### ^1^Center of Education, Research & Simulation Enhanced Training (CRESENT), King Fahad Medical City, Riyadh, Saudi Arabia; ^2^Department of Medical Education, College of Medicine, King Saud bin Abdulaziz University for Health Sciences, Riyadh, Saudi Arabia

**Background:** Non-technical skills (NTS) have been incorporated into many undergraduate medical schools’ curricula and identified as important skills for healthcare workers. The best available evidence showed that the communication skills training should use experiential methods. Simulation-based scenarios followed by debriefing will give the chance for healthcare workers to have the experience followed by feedback.

**Objectives:** The aim of this study was to assess if team debriefing after a simulation-based scenario improved NTS; and to identify the elements of debriefing that lead to improvement of these skills.

**Methods:** The study design was randomized, pre-test post-test, control group, experimental design. The sample size was calculated using Openepi sample size calculation tool. The teams were randomized into two arms, the debriefing group and the control group. Each team attended a 4-hour simulation activity consisting of an interactive lecture about NTS followed by two high fidelity scenarios on myocardial infarction resuscitation and anaphylaxis resuscitation. The order of the two cases in each course was variable based on concealed randomization. All teams in the intervention group received the debriefing immediately after the first case by a certified simulation educator. All the events within the simulation centre were video recorded. Four performance raters blinded to the grouping and the order of the cases reviewed and rated all 66 videos using the global rating scale of TEAM assessment tool [1]. The rating scale for the 11 elements ranged from 0 to 4. The main variables included are: leadership, teamwork, situation awareness and task management.

**Results:** A total of 136 nurses and 34 physicians from the emergency department at King Fahad Medical City (KFMC) were randomized into 34 teams. Each team consisted of 4 nurses and a physician. One group was excluded from the data analysis due to an issue with video recording. Debriefing after the simulation-based scenario significantly improved NTS TEAM assessment total mean score in the study group compared to controls, 4.8 points with *p* = 0.015 vs. 3.9 points with *p* = 0.11 respectively (Fig. 1). The detailed scores are shown in Table 1.

**Conclusion:** Simulation based scenario followed by debriefing and feedback is an effective teaching tool to improve NTS. The improvement is mainly noted in leadership, communication and team work. Future studies are needed to explore how long those skills are maintained and their application to real practice.

**Fig. 1 (abstract A17).**
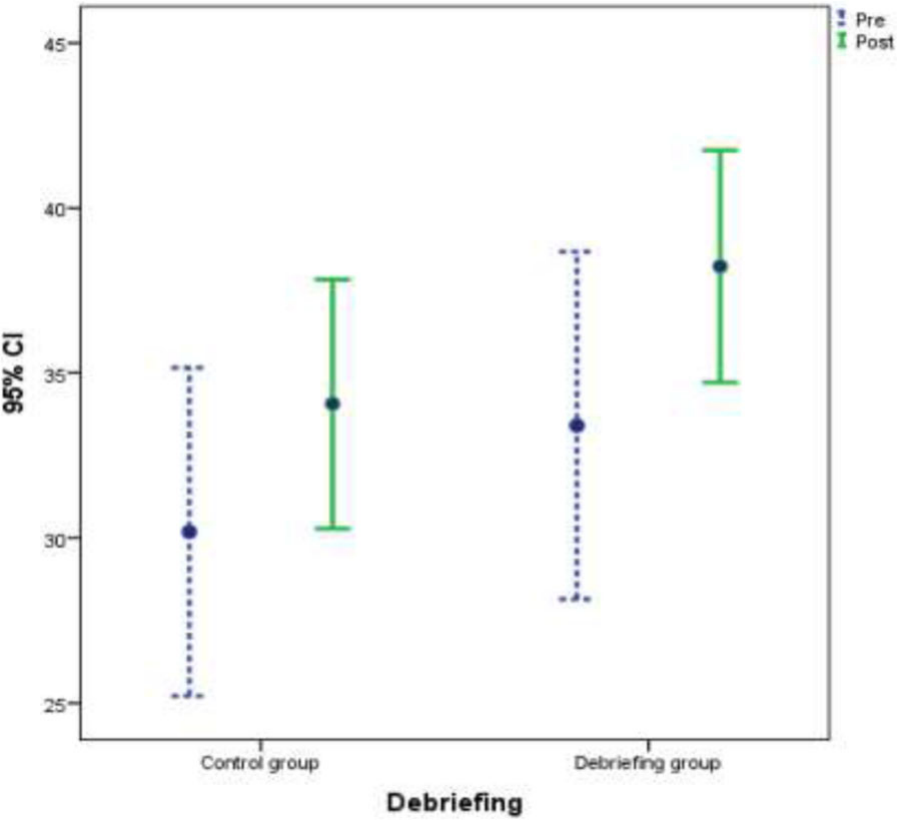
Pre and post-performance mean scores in the two study groups

**Table 1 (abstract A17).**
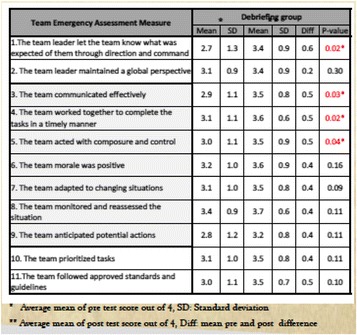
The summary of average mean score for pre-test and post-test for each TEAM item

**Reference**

1. Cooper, S., Cant, R., Connell, C., Sims, L., Porter, J., Symmons, M., Nestle, D., Liaw, S. (2016). Measuring teamwork performance: Validity testing of the Team Emergency Assessment Measure (TEAM) with clinical resuscitation teams. *Resuscitation*, 97-101. 10.1016/j.resuscitation.2016.01.026.

## A18 Impact of simulation-based medical education on performing infant lumbar puncture among pediatric residents

### Osama Obaid, Walaa Almaghrabi

#### Department of Pediatric, Hera General Hospital, Makkah, Saudi Arabia

**Background:** Infant lumbar puncture (ILP) is one of the most important skills for every pediatrician to master. Simulation-based medical education (SBME) is new way to train medical student and pediatric resident on various procedures.

**Objective:** Our study aims to investigate the impact of one session of SBME on performing ILP on pediatric residents.

**Methods:** This was an observational study where pediatric residents were exposed to one session of SBME by an expert instructor. A post exposure survey was collected and analyzed.

**Results:** A total of 100 pediatric residents participated of training levels PGY2 (54%), PGY3 (10%) and PGY4 (36%). There were 45 % male and 54 % female residents. Around 72% of the pediatric residents felt confident to do ILP. All were able to identify landmarks for ILP correctly. Only 27 % of our surveyed residents thought SBME improved their success rate in ILP procedure and 57% were aware of the ILP critical checklist.

**Conclusion:** One session of SBME to teach ILP increased the confidence of pediatric residents. However, only a few learners thought it improved their skills. More research is needed to study impact of SBME on skill acquisition among residents in training.

**References**

1. Kessler, David O., et al. “Interns’ success with clinical procedures in infants after simulation training.” Pediatrics (2013): peds-2012.

2. Kessler, David, et al. “Impact of just-in-time and just-in-place”.simulation on intern success with infant lumbar puncture Pediatrics (2015): peds-2014.

## A19 Simulation technologist: a new addition to the healthcare workforce in Saudi Arabia – proposed job description & career pathway

### Mohamad AlAmar^1^, Jalal AlFroukh^1^, Faisal AlSalem^2^, Hani Lababidi^1^

#### ^1^CRESENT, King Fahad Medical City, Riyadh, Saudi Arabia; ^2^Human Capital Administration, King Fahad Medical City, Riyadh, Saudi Arabia

**Background:** The growth of health simulation in Saudi Arabia necessitated skilled technical staff to assist educators in the design, operation and maintenance of appropriate simulation technologies. Simulation technologists or simulation operations specialists have diverse backgrounds with different educational levels.

**Objective:** The aim of this project is to propose a job description and career pathway for simulation technologists.

**Methods:** A review of the literature was made to look for publications regarding characteristics, job descriptions and career pathways for simulation technicians, operation specialists and/or technologists. After appraising various models and experiences and the benchmark in the market, a draft job description was made in accordance to the rules and regulations of the Ministry of Health (MOH) and Ministry of Civil Service in Saudi Arabia. The simulation team worked with experts from talent acquisition and human capital departments at KFMC to come up with a proposed career pathway using a three-step modified Delphi method to establish consensus.

**Results:**

- Terminology: The consensus of the simulation and human resources experts is to use the term “simulation technologist” over “simulation technician” or “simulation operation specialist”. The “technologist” term is more inclusive than technician, and “specialist” has specific connotation in the MOH lists for medical career paths.
- Educational background: A degree (diploma, bachelor, masters or PhD) in nursing, respiratory therapist, emergency medical services, allied health, biomedical sciences or engineering or information technology.
- Job description:
  - Prepare setup, allocate and provide all requirements for skills training activities and OSCE exams.
  - Operate the training tools, models and simulators.
  - Coordinate with course directors and instructors to execute training plans.
  - Demonstrate how to use training modalities and educational instruments for all instructors and self-training learners.
  - Make recommendations for the equipment and materials required for simulation-based sessions.
  - Participate in related research and innovations.
  - Allocate facility and training tools with best utilization of space and equipment.
  - Maintain stock levels and equipment inventory to meet work needs.
  - Maintain the simulation equipment to a high functioning standards.
  - Record data for all simulation activities inside and outside the center.
  - Manage, maintain and record all loaning procedures.
  - Maintain and promote strict confidentiality about performances, courses’ content, persons and OSCE’s.
  - Follow all relevant simulation center policies, processes, standard operating procedures and instructions so that work is carried out in a controlled and consistent manner.
  - Contribute to the identification of opportunities for continuous improvement of systems, processes and practices taking into account leading practices, improvement of business processes, cost reduction and productivity improvement.
  - Promote the implementation and adherence to policies, processes and operating procedures to others within the mother institution.
- Career Pathway: the proposed career path is presented in the figure below.

**Fig. 1 (abstract A19).**
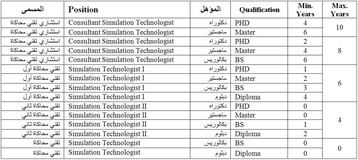
Career pathway of simulation technologists

**Conclusion:** The simulation technologist is a new addition to the healthcare workforce in Saudi Arabia. We hope it will be officially recognized by the Saudi Commission of Health Specialties (SCFHS) in the near future.

## A20 The elements of simulation system integration: the CRESENT Model

### Hani Lababidi, Shadi AlMozainy, Abdullah AlMarshed, Osama AlZoraigi, Abdulrahman AlSabbagh

#### CRESENT, King Fahad Medical City, Riyadh, Saudi Arabia

**Background:** Simulation system integration can be defined as consistent, planned, collaborative, integrated, and iterative application of simulation-based assessment, research, and teaching activities with systems engineering, and risk management principles to achieve excellent bedside clinical care, enhanced patient safety, and improved outcome metrics across the healthcare system(s) [1].

**Objective:** The aim of this project is to develop a practical model for simulation system integration.

**Methods:** A panel of 5 experts in health simulation used a three-step modified Delphi method to establish consensus. The first round was a face-to-face meeting where the essential steps of the simulation system integration were agreed. Round 2 consisted of defining three items for each step. The members of the expert panel marked “agree” or “disagree” beside each statement, and provided comments. Eighty percent agreement was used to determine acceptance or rejection of a statement. Statements that did not meet consensus from round 2 were emailed to all 5 members. In round 3, the experts used the same voting method, but with the knowledge of the scores and comments.

**Results:** The developed simulation system integration model is a seven step model that follows the acronym of CRESENT. The first step “**C**larify” involves: 1) Capture the needs, problems or concerns. 2) Characterize the impact and priority of the problem. 3) Classify involved stakeholders. The second step “**R**eview” involves: 1) Review available data/metrics on the concerns. 2) Relate to planned or ongoing projects on the concerns. 3) Recommend metrics that can be tracked. The third step “**E**xamine” involves: 1) Employ root-cause analysis, 2) Evaluate potential causes and 3) Estimate areas of simulation intervention. The fourth step “**S**imulate” involves: 1) Set system modeling (2) to simulate vital characteristics. 2) Select simulation modalities and environments. 3) Sketch simulation-based interventions. The fifth step “**E**xecute” involves: 1) Employ simulation-based interventions. 2) Extend simulation intervention/model to involve all stakeholders. 3) Evaluate as you simulate. The sixth step “**N**otify” involves: 1) Navigate detailed report of outcomes. 2) Name stakeholders to receive the report. 3) Note feedback from stakeholders. The seventh step “**T**rack” involves: 1) Tune new data to metrics that can be tracked. 2) Transform metrics/KPIs to a dashboard. 3) Troubleshoot deviation from norms.

**Conclusion:** The CRESENT model provides a well-defined stepwise approach for simulation system integration.

**Reference**

1. William Dunn, Ellen Deutsch, Juli Maxworthy, Kathleen Gallo, Yue Dong, Jennifer Manos, Tiffany Pendergrass, Victoria Brazil. Systems Integration. In: Adam I. Levine, Samuel DeMaria Jr., Andrew D. Schwartz and Alan J. Sim (eds.) The Comprehensive Textbook of Healthcare Simulation 10.1007/978-1-4614-5993-4 © Springer Science + Business Media New York 2013.

## A21 The elements of eye tracking that differentiate user experience on laparoscopic virtual reality simulator

### Nada Almohaimeed^1,2^, Hani Lababidi^3^, Mohammed Alamar^3^, Fazale-e-Amin^1^, Areej Al-Wabil^4^

#### ^1^College of Computer and Information Sciences, King Saud University, Riyadh, Saudi Arabia; ^2^Department of Natural Sciences and Engineering, King Saud University, Riyadh, Saudi Arabia; ^3^CRESENT, King Fahad Medical City, Riyadh, Saudi Arabia; ^4^Center for Complex Engineering Systems, King Abdulaziz City for Science and Technology (KACST), Riyadh, Saudi Arabia

**Background:** Virtual reality (VR) surgical simulation provides safe and realistic learning environment and can improve trainees’ skills and performance [1]. Eye movements’ measurements such as *fixation* (moments when eyes are relatively stationary, taking in or encoding information) and *saccade* (quick eye movements occurring between fixations) can reveal the amount of information processing applied to interface elements by individuals [2].

**Objective:** The aim of this study is to determine what elements in eye tracking during VR surgical performance can segregate novices from experts.

**Methods:** The subjects performed the clipping module (Fig. 1) on LapVR surgical simulator (CAE Healthcare). This skill required appropriate traction to correctly place four clips to stop blood flow, and then cut between the clips. Subjects can navigate and change the angle of the view by moving the camera handle. Eye movements were recorded via Tobii X120 (Stockholm, Sweden). A full HD video recording was captured via a Logitech C920 HD webcam (Fig. 2). The electrodermal activity (EDA) signal was obtained for the whole session including the baseline for each participant.

**Results:** A total of 19 participants, 6 experts (E) and 13 novices (N) were included. Mean age 34.8+7.5 years (range: 25-50). There was no significant effect on the total fixation duration (E=126 seconds and *N* = 144, *p* = 0.115). No significant differences in the mean fixation duration was observed (E=444ms ±100 vs. *N* = 368ms±121, *p* = 0.240). There was no significant difference in the fixation rate (E=121±28 vs. *N* = 122±18, *p* = 0.873). By investigating areas of interest (AOIs), novices fixated longer on the tools menus which means that they had greater uncertainty selecting the appropriate tool than experts. Gaze-plots and clusters revealed that the intensity of fixations and the spatial distribution of fixations are different among the two groups where novices show more of scattered saccades. Experts performed the task significantly faster than novices (E=146 ±13 vs. *N* = 207±64 seconds, *p* = 0.005). EDA level for novices was significantly higher than experts during the performance, *p* = 0.009. Aggregate gaze data showed a contrast in the spatial and temporal features of gaze-plots of experts when compared to novices. The number fixations, intensity of fixations and the spatial distribution of fixations were higher for novices than experts. When comparing clusters, novices tend to adjust camera handle to show the vessel in closer angle to their dominant hand, whereas experts place it to a wide angle showing the vessel in the middle of the screen. Figure 4 shows the number of mistakes done in each performance measure by each group. Experts made an average number of mistakes of 0.83, whereas novices made an average of 3.92.

**Conclusion:** The performance results of our experiments showed that experts were quicker and generally exhibited fewer errors than novices. The eye gaze analysis did not show marked differences between experts and novices. However, spatial density of fixation was different.

**References**

1. Seymour, NE; et al. Virtual reality training improves operating room performance: results of a randomized, double-blinded study. Annals of Surgery 2002;236(4):458-464

2. Ball, A.P.a.L.J., Eye Tracking in Human-Computer Interaction and Usability Research: Current Status and Future. Prospects, Chapter in C. Ghaoui (Ed.): Encyclopedia of Human-Computer Interaction. Pennsylvania: Idea Group, Inc, 2005.

**Fig. 1 (abstract A21).**
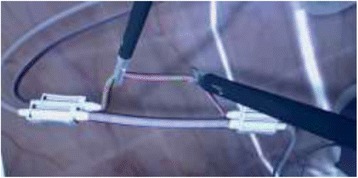
The clipping VR module

**Fig. 2 (abstract A21).**
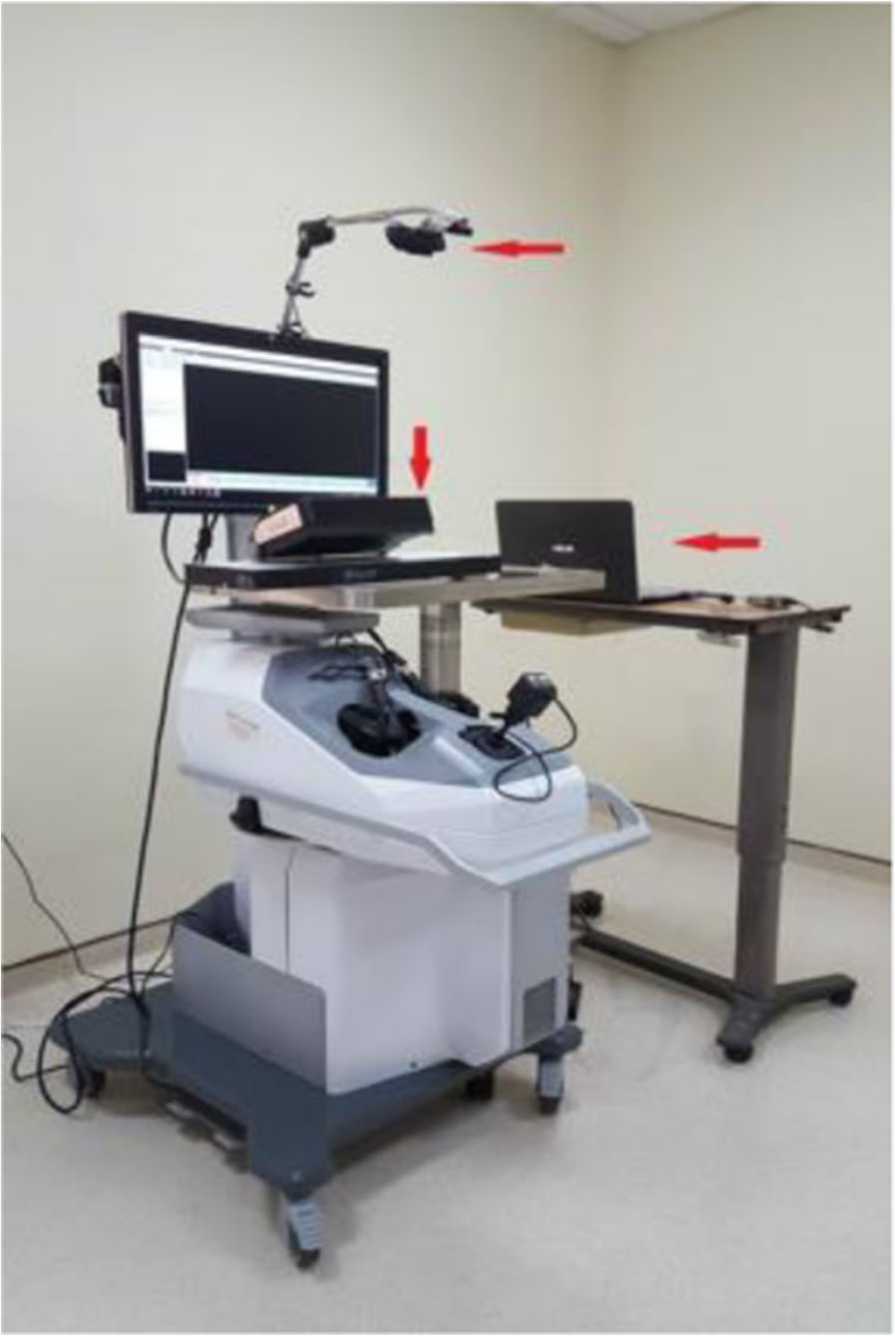
Eye tracking and HD camera mounted on CAE LapVR

**Fig. 3 (abstract A21).**
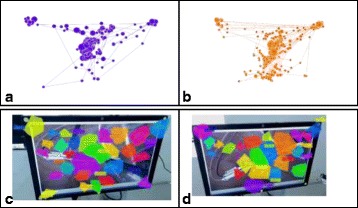
Gaze plots for expert (**a**) and novices (**b**), clusters for expert (**c**) and novices (**d**)

**Fig. 4 (abstract A21).**
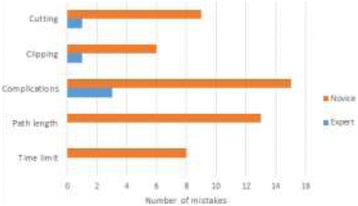
Number of mistakes per each category

## A22 Assessment of nurses’ knowledge of basic life support resuscitation guidelines in a tertiary hospital, southern region of Saudi Arabia

### Mesfer Algashanin

#### Abha Psychiatric Hospital, Abha, Saudi Arabia

**Background:** Studies have shown that there is an increase in survival rate from cardiac arrest (CA) when the knowledge and skills learnt in Basic Life Support (BLS) courses are administered [1]. Nurses in Saudi Arabia are the first responders when patients experience CA. It is mandated that they obtain the BLS certificate to work in any healthcare facility [2].

**Objectives:** The study aimed to assess the knowledge of BLS guidelines and the relationship between nurses’ knowledge of BLS and their professional profile.

**Methods:** The study is a non-experimental, quantitative, cross-sectional design. Sampling method was a non-probability, purposive. The data collection tool was a questionnaire. The study targeted all registered nurses in the hospital. The significance level was set at p <0.05, while the level of confidence was set at 95 per cent.

**Results:** A total of 172 nurses were included in the study, 32 (18.6%) got their BLS training via high fidelity simulation courses. They scored higher grades than others in each aspect of BLS knowledge. As the results of the Kruskal-Wallis test indicate that, the significance level computed for the assessment’s knowledge (*p* = 0.016), effective chest compression knowledge (*p* = 0.023), the effective cardiac defibrillation knowledge (*p* = 0.014) and the air way management knowledge (*p* < 0.005). Nurses aged 21-30 years (*n* = 58, 33.7%) scored higher grades than other age groups in the assessment’s knowledge (*p* = 0.014) and in correct chest compression (*p* = 0.021). Surgical nurses (*n* = 42, 24.4%) had a higher score in the BLS knowledge in comparison with nurses in the medical and outpatient departments. The significance levels computed for the assessment knowledge (*p* = 0.020), effective chest compression knowledge (*p* = 0.034) and the effective cardiac defibrillation knowledge (*p* = 0.023). Nurses (*n* = 40, 23.3%) who were updated on evidence-based nursing practices in BLS in the last 6 months were more knowledgeable in effective chest compression (*p* = 0.006) and the airway management (*p* = 0.025).

**Conclusion:** Nurses’ professional profiles appear to have an impact on their BLS knowledge. Using high fidelity simulation technique in BLS courses is the preferred choice when training the nurses [3]. A refresher evidence-based BLS course every six months will be beneficial for clinical nurses [4]. Nurses who work in the surgical department may be the best choice to be a resuscitation team member.

**References**

1. Aldawood, A. (2007). The outcomes of patients admitted to the Intensive Care Unit following cardiac arrest at a tertiary hospital in Saudi Arabia. Polskie Archiwum Medycyny Wewnetrznej, 117(11–12), 497–501. Retrieved from http://www.ncbi.nlm.nih.gov/pubmed/18363249.

2. AlYami, M. S., & Watson, R. (2014). An overview of nursing in Saudi Arabia. Journal of Health Specialties, 2(1), 10. JOUR.

3. Lucas, A. N. (2014). Promoting continuing competence and confidence in nurses through high-fidelity simulation-based learning. Journal of Continuing Education in Nursing, 45(8), 360–5. 10.3928/00220124-20140716-02

4. Cooper, S., McConnell-Henry, T., Cant, R., Porter, J., Missen, K., Kinsman, L., Scholes, J. (2011). Managing deteriorating patients: registered nurses’ performance in a simulated setting. The Open Nursing Journal, 5(1). JOUR.

## A23 Simulation-based system integration in commissioning new Emergency Department

### Shadi AlMoziny, Abdullah AlMarshad, Amani Azizalrahman, Abdulrahman Sabbagh, Jalal AlFroukh, Mohamad AlAmar, Hani Lababidi

#### Center of Education, Research & Simulation Enhanced Training (CRESENT), King Fahad Medical City, Riyadh, Saudi Arabia

**Background:** There are integral faults that can be faced upon starting new departments in a healthcare facility. These errors can affect patient safety and lead to medical errors. Medical simulation can be used to diagnose these potentials errors so the can be corrected prior to commissioning of the new service.

**Objectives:** The objective of this study is to utilize simulation-based system integration methodology to identify areas of improvement prior to starting a new Emergency Department (ED) at King Fahad Medical City (KFMC).

**Methods:** A new ED was constructed to improve emergency medicine services and meet the expanding demands at KFMC. The new ED has a capacity of 50 beds with complete radiology, pharmacy and laboratory services. Prior to commissioning, a simulation drill was performed to identify latent safety threats and potential weaknesses and potential errors in the system. A total of 12 scenarios were written and 3 SPs and 2 high fidelity manikins were utilized in the drill. Complete moulage was performed on SPs and manikins as needed. The outcome measures were classified as major and minor. Major weaknesses are defined as defects that may compromise patient safety, while minor ones are those related to resource allocation, patient flow and other administrative issues not affecting the quality of care and patient safety. A team of 3 ED physicians with special expertise in simulation recorded the observations.

**Results:** A total of 80 observations were recorded. These were 5 majors and 75 minors. Example of major weaknesses were unavailability of crash carts in some areas, incomplete training of the staff on the new cardiac monitors and technical problems in the intradepartmental communications. Major observations were all corrected before actual ED operation, while minor observations were corrected before or shortly after ED operation.

**Conclusion:** Simulation-based system integration is an imperative tool to early identify latent safety threats and thus can improve patient safety.

## A24 CRESENT System Integration Committee: an innovative approach to system integration in healthcare simulation

### Shadi Almoziny, Hani Lababidi

#### Center for Research, Education & Simulation Enhanced Training (CRESENT), King Fahad Medical City, Riyadh, Saudi Arabia

**Background:** The involvement of medical simulation within any healthcare system is rarely integrated into the whole healthcare delivery process. Rather, medical simulation is usually involved as a sporadic, unintegrated, unplanned and inconsistent intervention.

**Methods:** The System Integration Committee, under the Center for Research, Education & Simulation Enhanced Training (CRESENT), King Fahad Medical City (KFMC), is formed to apply consistent, planned, collaborative and integrated interventions, in conjunction with systems engineering and risk management, to address KFMC healthcare system issues and needs and achieve excellent clinical care, enhanced patient safety and improved outcome metrics across the healthcare system. The committee is led by a chairperson who is a simulation expert with fellowship training in medical simulation. Objective data and metrics about KFMC healthcare system performance are collected by concerned groups, departments or administrations that includes: Corporate Planning & Development Administration, Quality Management Department, Risk Management Department, Continuing Professional Development Administration, Executive Nursing Administration. The strategy that was followed at CRESENT regarding System Integration was to bring the leaders at KFMC around the same table as members of CRESENT System Integration Committee. The committee membership requires the members to be in higher leadership positions within the healthcare system at KFMC because they are looking at the bigger picture of the healthcare system and can prioritize the needs.

**Results:** The committee agreed to prioritize its work to address the High Volume-High Impact issues that are well documented and tracked through objective metrics. Also, the committee agreed to address other issues whenever they arise based on their urgency and impact. Patient handover was identified as one of the major High Volume-High Impact issues that need to be addressed. The simulation program at CRESENT is now part of the iSBAR Handover Project and it is involved in multiple steps within the project that are applicable to simulation.

**Conclusion:** The committee brought CRESENT system integration initiative to a higher level, i.e. the Quality Council, which will support the execution of the committee recommendations. The committee represents an organized bi-directional feedback loop between CRESENT and the healthcare system at KFMC to help to integrate simulation programs within the whole KFMC healthcare system.

**References**

1. William Dunn, Ellen Deutsch, Juli Maxworthy, Kathleen Gallo, Yue Dong, Jennifer Manos, Tiffany Pendergrass, Victoria Brazil. Systems Integration. In: Adam I. Levine, Samuel DeMaria Jr., Andrew D. Schwartz and Alan J. Sim (eds.) The Comprehensive Textbook of Healthcare Simulation 10.1007/978-1-4614-5993-4 © Springer Science + Business Media New York 2013.

2. National Academy of Engineering and Institute of Medicine. Building a better delivery system: a new engineering/health care partnership. Washington: National Academies Press; 2005.

## A25 Cadaveric workshops in residents surgical training - which cadaver to select and for what types of procedures?

### Mai Ahmed Banakhar

#### King Abdulaziz University, Jeddah, Saudi Arabia

**Background:** The aim of study was to assess the use of cadavers and examine their suitability for surgical procedures training in urology. Our hypothesis was all types of cadavers are suitable for surgical procedures training in urology, this hypothesis will be rejected at p value less ≤ 0.05.

**Methods:** We conducted a randomized single blinded study on cadaver at King Abdulaziz University Urology Department in collaboration with Skill Center and Anatomy Department of the College from March 2014 to March 2017. We developed two different cadaveric surgical training preparations: fresh frozen versus perfused cold non-frozen cadavers. A total of 4 cadavers in each groups were prepared and used for this study. Urology procedures included endo-urology, abdominal surgery both retroperitoneal and transperitoneal, and genital reconstructive procedures. Residents were randomly allocated to different cadaveric preparations and underwent four surgical training days under supervision of a urologist in each arm. At the end of each day, trainees were requested to complete a questionnaire assessing the quality of cadaveric-based learning in their course.

**Results:** A total of 44 residents were trained in the courses (22 in each arm). Endo-urology procedures were difficult in both preparations frozen and cold perfused cadavers (percutaneous nephrolithotomy and uretroscopy). Genital operations where feasible on both cadaveric preparations equally *p*-value= 0.3. Abdominal procedures were difficult on fresh frozen cadavers because of fermentation and bad odor in comparison to perfused cadaver, *p*-value= 0.023. At the fourth day, fresh frozen cadavers were not suitable for use, while cold perfused were stored and used up to six months in other courses.

**Conclusion:** Cold perfused cadavers are superior to fresh frozen cadavers in urology surgical training. We recommend it for abdominal, pelvic and genital operations.

## A26 Innovation in wound management utilizing simulation-based training

### Alla BaMohammad, Sharifah Mohidin, Shiney John, Aishatu Alhassan, Shyla Frank, Beena Joshy, Nerissa Yutuc, Teofista Roxas, Bakrya Alrashdi, Souad Almelki, Rubyrosa Mirarza, Ghada Abu Alnaja

#### King Abdulaziz University Hospital Education, Training & Research Unit, Jeddah, Saudi Arabia

**Background:** King Abdulaziz University Hospital (KAUH) Education, Training & Research Unit plays a vital role in ensuring nurses to be constantly updated with evidence-based practice and providing high quality of care and patient safety. A learning needs survey identified that a wound management workshop was one of the priorities required by nurses, both clinical and managerial. Since, simulation-based training was recognized as the best method, it was incorporated in this workshop to develop nurses’ knowledge, skills and attitudes, applying real scenario in resolving practical dilemmas whilst ensuring patient safety and diminishing errors.

**Objectives**: The aim of this presentation is to explore the effectiveness of simulation-based training in enhancing nurse’s wound management knowledge, skills and attitudes from an holistic perspective and building a cohesive team.

**Methods**: A six-hour workshop was developed with integration of multi methods of teaching including lectures, simulation-based training with demonstration & return demonstration. Simulation was used in teaching of wound assessment, diabetic and pressure ulcers, VAC machine and aseptic technique. A post-training survey assessed the effectiveness of the workshop.

**Results**: A total of 220 nurses attended 11 workshops. The post-training survey showed nurses’ overall satisfaction of the workshop was *very good* to *excellent* (mean=96%). They found the effectiveness of integrating simulation-based training was satisfactory. The nurses found that simulation enhanced their clinical practice (mean 98%) and enable changes to better practice (mean 95%). Themes in the qualitative data were; practicability, valuable & updated knowledge, recommendation for all other nurses to attend. Respondents recommended increasing the length of time for the workshop.

**Discussion:** Respondents reported benefits of attending the workshop. This is further supported by a previous an observational cross-sectional study among 41 nurses comparing participants who attended wound management course (*n* = 20; 48.8%) and nurses who did not attend this course (*n* = 21). The chi-square test revealed a significant difference in dressing done with completely aseptic techniques among participants who attended the wound management course (*p* = 0.02). In general, among all nurses the mean score for nurses’ adherence to correct wound management techniques was 77.6%. Compliance was lower in the pre-phase, confirming the dressing order: *n* = 27 (66.7%), conducting client verification *n* = 29 (71.8%), and explaining the procedures to the client *N* = 24 (59%). Compliance was higher during the performance phase (*n* = 36; 90%). However, the least compliance was shown in the post-phase in the area of educating patients and family by only 33.3% (*n* = 13).

**Conclusion**: Integrating simulation-based teaching in a wound management workshop was found to be effective and was reported to improve clinical practice. The simulation has been integrated in other KAUH nursing workshops and courses based on these findings.

**Reference**

1. Alla Ba Mohammad. The effect of wound management course on nurses, an observational study. 2016, unpublished work.

## A27 Improving medication administration safety for nurses through simulation-based competency

### Sabo Yusuf Machudo, Kholoud Abdullah, Badria Abdulaal, Sonymol Mattam, Florencia Leyba, Hauwa Ibrahim

#### King Abdulaziz University Hospital, Jeddah, Saudi Arabia

**Background:** Medication management is one of the common procedures performed by nurses in hospitals. Most, if not all, patients admitted to a healthcare facility receives medication during their hospitalization. It is a high volume procedure performed by nurses where safety may be compromised. In King Abdulaziz University Hospital (KAUH) medication administration errors and near misses are a growing challenge. The need for solutions is imperative for patient safety. Nurses, being in the forefront to prevent potential administration medication errors need to be competent, confident and skilled.

**Objectives:** The aim of this project is to improve medication administration skills of nursing staff in KAUH using simulation to enable them to meet expected competency for patient safety.

**Methods:** The project was carried out from January to December 2017 in KAUH Clinical Skills and Simulation Center. The simulation-based competencies program consists of eight workshops of four-hour duration. It included a pre-test, lecture discussion on relevant KAUH medication policies, simulation-based competencies using task training mannequin and available equipment in the center related to the administration of various medications via different routes. From the eight workshops, four groups were randomly selected using snowballing sampling method to analyze the average pre- and post-tests data and participants’ evaluations.

**Results:** Of the 922 nurses who are providing clinical care at KAUH, 380 (38%) attended the safe medication practice program. In the evaluation, 78% reported that the simulation was excellent, 14% as very good and 8% reported as good. The participants recorded higher scores in the post-test with mean difference of 15.5% (pre-test: 74.8% and post-test: 90.3%, p <0.01). The rate of medication administration errors in the hospital, as reported by the King Abdulaziz Hospital Quality department, was 0.09 errors per patient days in 2016 and it dropped to 0.03 errors per patient days in 2017, a reduction rate of 67%.

**Conclusion:** The findings indicate that participants have benefitted from the safe medication practice program. It has increased nurses’ knowledge, skills, ability and confidence in medication administration practices which contributes to reduction in errors thereby improving patients’ safety.

